# Porewater salinity in a southeastern United States salt marsh: controls and interannual variation

**DOI:** 10.7717/peerj.5911

**Published:** 2018-11-08

**Authors:** David Miklesh, Christof Meile

**Affiliations:** Department of Marine Sciences, University of Georgia, Athens, GA, USA

**Keywords:** *Juncus roemerianus*, Modeling, Porewater, Simulation, Salt marsh, *Spartina alterniflora*, Salinity

## Abstract

In coastal marsh ecosystems, porewater salinity strongly affects vegetation distribution and productivity. To simulate marsh porewater salinity, an integrated, spatially explicit model was developed, accounting for tidal inundation, evaporation, and precipitation, as well as lateral and vertical exchanges in both surface waters and the subsurface. It was applied to the Duplin River marsh, Sapelo Island, USA, over a 3-year period, which covered both drought and wet conditions. Simulated porewater salinity in the low and high marsh correlated with Duplin River salinity, with evapotranspiration and precipitation leading to substantial variations in porewater salinities across seasons, in particular in the high marsh. The model revealed substantial interannual variability in marsh soil conditions, and—due to its process-based approach linked to external forcings—can be used to explore effects of sea level rise and changes in hydrological forcings on marsh soil conditions.

## Introduction

Salt marshes experience regular tidal inundation with brackish or oceanic water. They are prevalent in low-energy wave environments typical of regions between barrier islands and the coast (Wiegert & Freeman, 1990). Intertidal salt marshes are some of the most productive ecosystems on Earth, providing many important ecosystem services, which include: carbon and nitrogen sequestration and transformations, the provision of habitats, reduction of erosion, and mitigation of hurricane impacts on coastal infrastructure (Craft, Broome & Seneca, 1988; Loomis & Craft, 2010; Barbier et al., 2011; Fagherazzi et al., 2013; Kirwan & Megonigal, 2013). Understanding what controls the ability of a marsh to provide these services is thus of broad societal relevance.

In the Southeastern United States, salt marshes are dominated by *Spartina alterniflora*, with growth forms varying with elevation (Wiegert & Freeman, 1990). Above the *Spartina alterniflora-*dominated low marsh, several other plant species can be found, including *Juncus roemerianus, Sarcocornia* spp. (formerly *Salicornia virginica*)*, Batis maritima, Distichlis spicata*, and *Borrichia frutescens* (Hladik, Schalles & Alber, 2013). In the last decade, several studies have focused on the interplay between soil conditions, water flow, and vegetation zones in salt marshes. Moffett et al. (2012) demonstrated that ecohydrological zones developed as a result of a combination of soil properties, evapotranspiration (ET), and local topography, along with spring-neap tidal inundation variability. This concept was further supported by observations in Wilson et al. (2015) that showed distinct hydrologic flow patterns in *Spartina alterniflora*, *Sarcocornia* spp*.,* and *J. roemerianus* zones—net downward flow in tall *Spartina alterniflora* zones, periods of net upwards flow in short *Spartina alterniflora* zones, and upward flow of brackish water in *Sarcocornia* spp. zones and fresh water in *J. roemerianus* zones during neap tides. These observations were then related to marsh stratigraphy, which showed higher conductive sandy soils underlying low permeability marsh soils. Using a subsurface flow model of a marsh transect, Xin et al. (2017) showed that density-driven flow mitigated salt accumulation via ET in the marsh interior, and demonstrated the dominant effects of ET and rainfall on low permeable marsh soils. Thus, while inundation water salinity and rainfall are major determinants for soil salinity in intertidal settings (De Leeuw et al., 1991), porewater salinity is also affected by—and potentially impact—subsurface flow (Xin et al., 2017; Shen et al., 2015; Wilson et al., 2015; Moffett et al., 2012, and references therein), and in turn influence vegetation distribution (Pennings, Grant & Bertness, 2005).

Marsh productivity varies with vegetation type (Giurgevich & Dunn, 1982) and thus the ecohydrological zone and its soil conditions. Furthermore, within a given vegetation type, marsh productivity is also strongly influenced by porewater salinity (Morris, 1995; Pennings, Grant & Bertness, 2005). O’Donnell & Schalles (2016) found a ∼35% decline in *Spartina alterniflora* aboveground biomass over a 28-year period on the Georgia coast, which correlated with drought frequency and severity, as well as other factors that are expected to affect porewater salinity. To quantify the effect of changes in hydrologic conditions on marsh soils, a model is presented to simulate porewater salinity across an intertidal salt marsh. It builds on work by Morris (1995) and Wang et al. (2007), who simulated porewater salinity in salt marshes. Our approach, which resolves the marsh surface as well as fluid above and below the marsh surface, expands on their work by considering lateral fluxes and seepage as well as vegetation zonation. Other processes considered include ET, precipitation, infiltration, drainage, and exchange between the sediment and overlying water. Utilizing a 3-year time series of measured forcings, combined with observational data on soil conditions, the model is used to (1) quantitatively identify the key processes that control the porewater salinity across a marsh gradient (low to high marsh), (2) determine the role subsurface processes have on porewater salinity, (3) study the seasonal and interannual variability in porewater salinity, and (4) establish a foundation to estimate vegetation responses related to soil salinity under changing external forcings.

## Methods

### Site description and porewater salinity measurements

**Figure 1:**
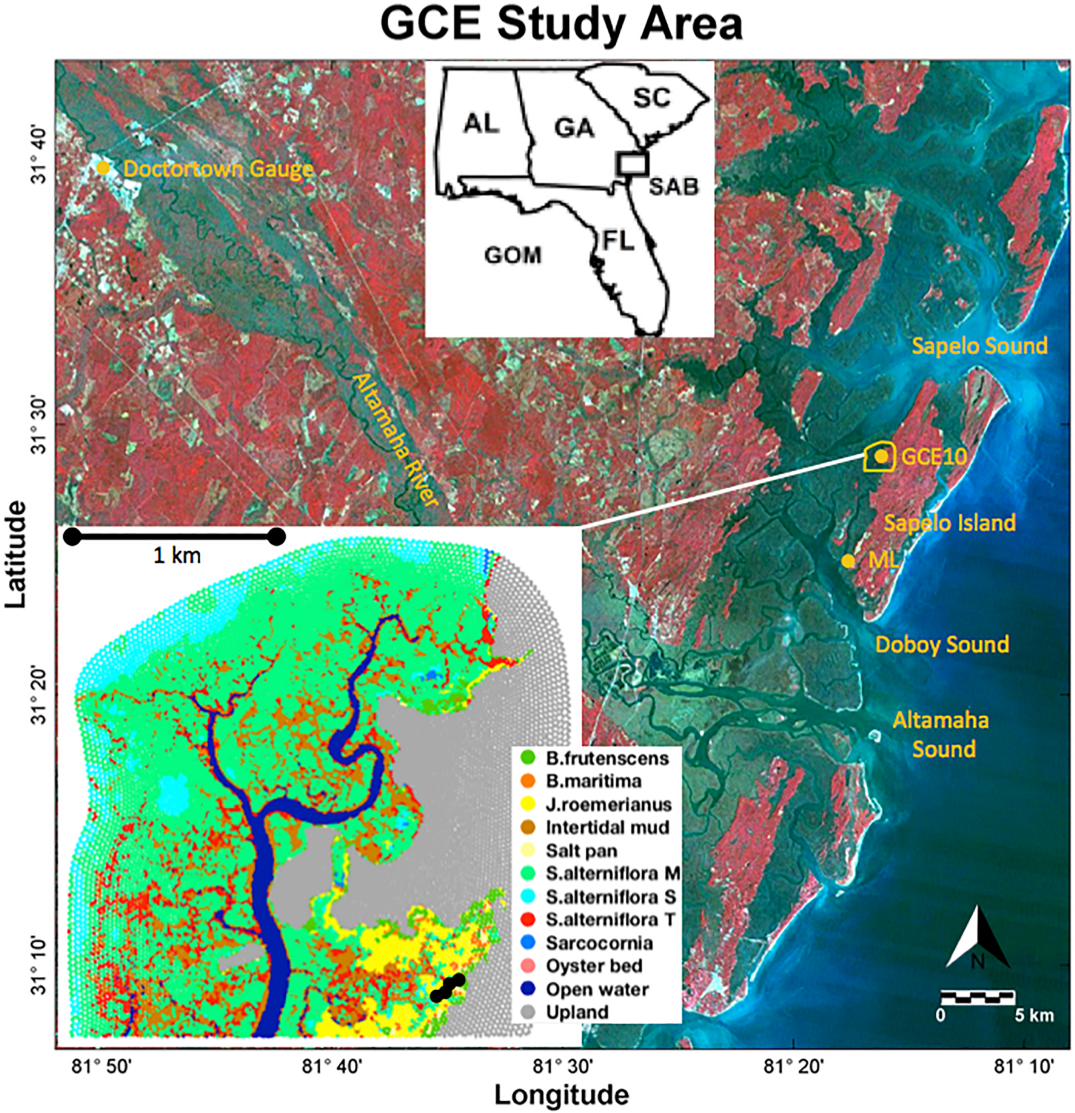
Study area. Landsat 5 color infrared satellite image with the study area outlined in yellow. Long-term monitoring stations GCE10 and Marsh Landing (ML) are marked with yellow dots. The inset shows the distribution of the vegetation classes; black dots near the lower right corner indicate the locations of observations. Landsat 5 image credit: USGS GloVis website at http://glovis.usgs.gov.

Utilizing the contextual data collected by the Georgia Coastal Ecosystems Long Term Ecological Research (GCE-LTER) project, the process-based soil model was applied to a marsh in the upper reaches of the Duplin River (Fig. 1) west of Sapelo Island at the coast of Georgia.

Located approximately 10 km north of the Altamaha River, the Duplin River is a large 12.5 km long tidal creek (Schutte et al., 2013), experiencing semidiurnal tides with a tidal range of 1.8 (neap) to 2.4 m (spring) (Ragotzkie & Bryson, 1955; Boon, 1975). Its salinity varies seasonally, ranging from 12 to 33 in the upper Duplin, and is influenced by Altamaha River discharge (Di Iorio & Castelao, 2013). Coastal precipitation, which averaged 85 cm/year between 2005 and 2012, is greatest during summer months when river discharge is lowest but does not correlate with Duplin salinity (Schutte et al., 2013).

Porewater salinities were obtained in an area encompassing a range of vegetation types (black dots in Fig. 1; see Supplement S4 for the data) in 16 sampling campaigns between July 2012 and November 2014. Porewater was collected from surficial soils at each sampling point using Rhizon Core Solution Samplers, pulling porewater from a depth of 10 cm using a hydrophilic porous polymer tube (Rhizosphere Research Products); salinity was measured with a handheld refractometer (Vee Gee STX-3).

### Model description

The soil model was based on mass conservation for water and salt, accounting for local sources and sinks, as well as subsurface and surface exchange processes (Supplemental Information S1). Individual marsh parcels were divided into above and belowground compartments. The belowground compartment encompasses the top 30 cm of sediment, while the vertical extent of the aboveground compartment varied with the height of the water column. The processes considered are outlined next.

#### Tidal flooding

Duplin River water level and salinity measurements from a hydrographic mooring located within the model domain (Station GCE10; Di Iorio, 2012a, 2013a, 2014a, 2014b) were used to describe tidal flooding. With natural levees separating the Duplin River from the low marsh, and small depressions scattered throughout the marsh, the tidal height at which a location floods does not necessarily correspond to the land elevation, and was determined by a flooding algorithm (Barnes, Lehman & Mulla, 2014; Supplemental Information S2).

#### Subsurface exchange

Groundwater exchange was comprised of lateral subsurface flow, vertical flow, and seepage. Flow was calculated using the Darcy equation:

(1) $$q =-{K \over {{\rm \rho} g}}\nabla {{P}}$$

where *q* is the Darcy velocity, *K* is the hydraulic conductivity [m/s], *ρ* is the fluid density [kg/m^3^], *g* is gravity [m/s^2^], and ∇*P* is the pressure gradient corrected for hydrostatic pressure [Pa/m]. Hydraulic conductivity was computed following Gardner (1958) as a function of the negative pressure head Ψ [m].

(2) $$K\left({{\Psi }} \right) = {K_s}{e^{{{\alpha \Psi }}}}$$

where *K_S_* is the saturated conductivity and the inverse capillary rise α [m] was set to 1 (Wilson & Gardner, 2006). Saturated water content and porosity were set to 0.425 (Gardner & Wilson, 2006), and residual water content was set to 0.255 (Hagens, 2010). At sites in the model domain, Schultz & Ruppel (2002) documented hydraulic conductivities that range from 1.6 × 10^−9^ to 2.3 × 10^−5^ m/s in marsh sediments, encompassing both very low permeability soils and highly permeable sands. Here, a saturated hydraulic conductivity of 2 × 10^−7^ m/s was applied to the low marsh, while a hydraulic conductivity of 2 × 10^−6^ m/s was imposed in the high marsh to reflect fine-grained mud mixed with sand. These values were comparable to large-scale infiltration estimates based on curve numbers and patterns of coastal rainfall as detailed in Supplemental Information S3.

Seepage, driven by the difference between porewater and atmospheric pressures, was allowed to occur when the marsh was exposed, and when the elevation difference between the centroids of two neighboring elements exceeded 30 cm. Following Morris (1995) and Wang et al. (2007), vertical drainage occurred when the tidal height was below the marsh platform elevation and greater than the field capacity—0.966 and 0.9 for the low and high marsh, respectively, which are within the range of values reported in Morris (1995) and Wang et al. (2007). To compute the drainage rate, pressure gradients were determined as the difference between the water table and the tidal elevation in the nearest creek divided by that distance. Saturated hydraulic conductivity was set to 2 × 10^−6^ m/s to reflect transport through a hydraulically conductive layer.

#### Atmospheric exchanges

Precipitation data was collected at a meteorological station located at Marsh Landing (in Fig. 1; Di Iorio, 2012b, 2013b, 2014c, 2015); hourly precipitation was computed from precipitation data measured at 15-min intervals. Precipitation was added to the belowground compartment until it became saturated, at which point precipitation was added to the aboveground.

Daily ET was computed from detailed meteorological inputs available (Di Iorio, 2012b, 2013b, 2014c, 2015) using the Penman–Monteith equation outlined in Allen et al. (1998), which has been shown to approximate ET in salt marshes (Hughes et al., 2001). Computed total daily ET rates were then distributed parabolically over a 12-h period (Morris, 1995). When parameterizing ET, differences between plant heights were considered. Using a vegetation map created from hyperspectral imagery and ground-truthing (Hladik, Schalles & Alber, 2013), each element of the mesh was assigned a surface coverage class (Fig. 1). Vegetated classes included *B. frutescens*, *B. maritima*, *J. roemerianus*, *Sarcocornia* spp., and short, medium, and tall *Spartina alterniflora*. Unvegetated classes included mud, salt pan, oyster shell, and open water. Mesh elements not contained within the boundaries of the hyperspectral map were assigned a class based on the elevation of the element centroid (Table 1), which is a strong predictor of vegetation classes (Hladik, Schalles & Alber, 2013). Each class was assigned an albedo—0.1 for vegetated classes, 0.08 for intertidal mud (Guarini et al., 1997) and oyster shell, and 0.059 for open water (annual average Fresnel albedo at 30° latitude; Cogley, 1979)—to compute the radiation balance, along with a vegetation height that was used to compute aerodynamic resistances (Table 1). Surface resistance was set to 0 for open water (Hughes et al., 2001) and saturated intertidal mud (Camillo & Gurney, 1986). After comparison with eddy covariance derived flux tower ET rates at our site (I. Forbrich, 2018, personal communication) a surface resistance of 70 s/m was used for all vegetation types, independent of tidal stage and season. Daily ET rates varied seasonally and ranged from approximately 0.25 mm/day minimum in the winter to 5 mm/day maximum in the summer for medium *Spartina alterniflora* and *J. roemerianus*.

**Table 1:** Vegetation information.

	Plant height (cm)	Elevation range (m MSL)
Tall *S. alterniflora*	150	0.023–0.767
Med. *S. alterniflora*	75	0.768–1.022
Short *S. alterniflora*	40	1.023–1.083
*J. roemerianus*	75	1.218–1.327
*Sarcocornia* spp.	40	1.122–1.152
*B. frutescens*	125	1.328–1.533
*B. maritima*	40	1.153–1.202

**Note:**

Vegetation height used to compute aerodynamic resistance for each class (Hladik, Schalles & Alber, 2013), and elevation range associated with a vegetation type for regions in which no areal survey data is available.

#### Salt exchange

The effect of salt excretion by plants (Bradley & Morris, 1991), bioirrigation (Meile, Koretsky & Van Cappellen, 2001), and diffusion were combined into a bulk term describing the exchange of salt between above and belowground compartments. *Spartina alterniflora,* a halophyte, has the ability to secrete salt ions from the shoots during transpiration (Bradley & Morris, 1991). Salt excretion is unidirectional and removes salt from the porewater to above ground, while diffusion is bidirectional and a function of the difference between above and below ground salt concentrations. However, Bradley & Morris (1991) reported that shoot excretion exports only 0.4–2.9% of soluble salt ions from the root zone per month, so salt exchange was implemented as

(3) $$J =-D{{{\bf{\Delta }}S} \over {{\bf{\Delta }}z}}$$

where *J* is the salt flux [kg/m^2^/h], *D* is an effective diffusion coefficient [m^2^/h], *S* is the salt concentration [kg/m^3^], and Δ*z* is the characteristic length, set to 0.01 m (Morris, 1995). The diffusion coefficient was set to 7.25 × 10^−6^ m^2^/h, as in Morris (1995) and Wang et al. (2007).

### Model implementation

The model used a 1-h time-step to resolve tidal flooding and ebbing and employed a triangular, unstructured grid (McKnight, 2016). The mesh covering the upper Duplin (Fig. 1) consisted of 17,833 nodes that made up 35,279 elements ranging in size between 48 and 423 m^2^, with smaller elements at topographic gradients. The topographic data was from a combination of light detection and ranging (LIDAR) flyovers and bathymetric soundings (Viso, 2011). The boundaries at the east, north, and west of the domain represent no flow catchment boundaries. The model domain extends southward into Doboy Sound, where subsurface flow is also set to 0. Note that only model results from the upper Duplin, more than seven km upstream of the southern boundary are analyzed, minimizing the effect of the boundary condition near the mouth of the Duplin on our analysis. This is motivated by the fact that the modeled tidal inundation is driven by water level data collected at GCE 10 (Fig. 1), which is an accurate approximation for that part of the domain, but less so for the lower reaches of the Duplin.

The model was implemented in MATLAB 2014b. For each time-step, the mass fluxes for water and salt were computed for tidal exchange, ET, precipitation, salt exchange, and groundwater flow. Then water volume, density, and salinity were computed for above and belowground compartments (see Supplemental Information S1). Simulations were run for 2012, 2013, and 2014, which represent dry, variable/wet and normal conditions based on the long-term Altamaha River discharge patterns. The simulation was started from July 2011, with a uniform salinity of 28, to ensure that the initial conditions did not affect simulations for 2012. For the sensitivity analysis, processes were perturbed within bounds representative of the uncertainties associated with them. For ET, precipitation, and tidal salinity simulations, the parameters were increased by 10%. Hydraulic conductivity was increased by an order of magnitude (reflecting the large yet poorly constrained uncertainty), salt exchange was doubled, mean sea level was increased by five cm, and field capacity was increased by 0.024 to reach 0.924 and 0.99 in high and low marsh, respectively. To determine the salinity response to parameter changes, model runs with and without parameter perturbations were compared for 2014, a year with average climatic conditions. The sensitivity was computed as the median percentage change relative to the simulated salinity:

(4) $$\% \Delta = Med\left( {{{S_i^{perturbed} - S_i^{baseline}} \over {S_i^{perturbed}}}} \right)*100$$

where subscript *i* indicates an element. Positive values mean that increasing a parameter will increase the porewater salinity (“positive effect”), while a negative value indicates a decrease in the porewater salinity compared to the baseline simulation (“negative effect”).

## Results

### Validation

**Figure 2:**
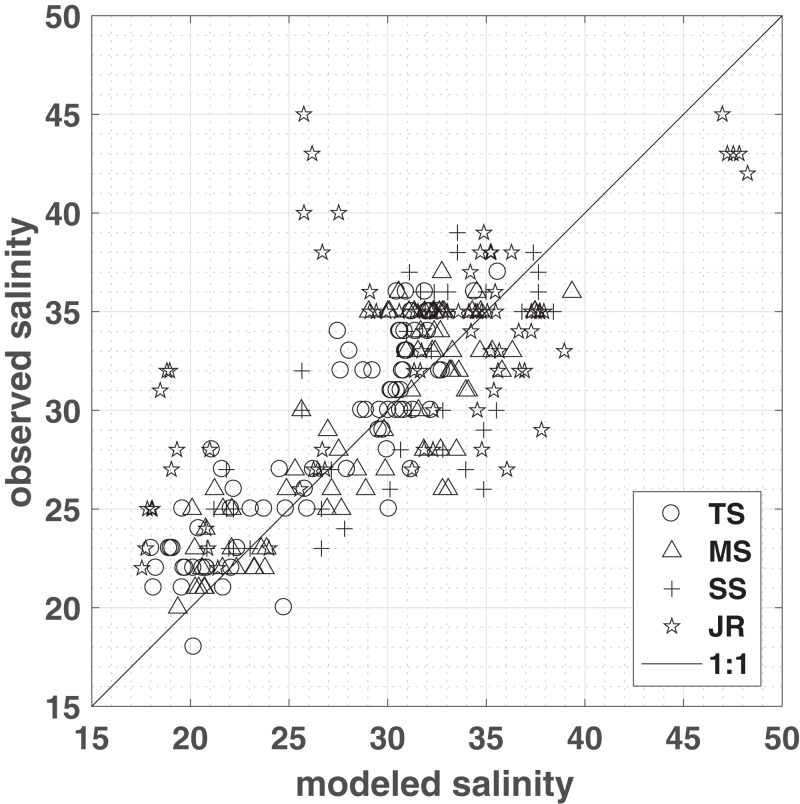
Model validation. Measured vs. modeled porewater salinities (means from sub-domain area encompassing observations matching both the vegetation type and the elevation of the observations within one cm), grouped by vegetation zones: tall *S. alterniflora* (TS), medium *S. alterniflora* (MS), short *S. alterniflora* (SS), and *J. roemerianus* (JR). A one-to-one line has been plotted for comparison. ZoneStats (sample size *n*, regression slope β, *r*^2^, RMSE): TS (80, 1.06, 0.83, 2.5); MS (78, 1.05, 0.77, 2.89); SS (48, 0.99, 0.64, 3.85); JR (80, 1.02, 0.62, 5.9).

Model simulations produce the spatial distribution of porewater salinities as well as water content over time (Figs. S2 and S3). To assess the performance, model simulations were compared to porewater salinities measured in 2012–2014 in four different vegetation plots, representing short, medium, and tall *Spartina alterniflora,* and *J. roemerianus* (Fig. 2). To account for differences in elevation—caused by topography that was not resolved in the model and inherent differences between LIDAR and RTK measurements (Hladik & Alber, 2012)—observations were compared to model salinities computed at locations that matched both the vegetation zone and the measured marsh elevation (±1 cm). For each vegetation class, a linear regression with slope β and a zero intercept was computed between the observation and the mean of the modeled salinities matching the above criteria (Fig. 2). The model successfully reproduced *Spartina alterniflora* observations in all seasons from 2012 to 2014, with a regression slope close to 1 and approximately ¾ of the variance explained—83%, 77%, and 64% for tall, medium, and short *Spartina alterniflora*, respectively. For *J. roemerianus*, the slope was approximately 1, with 62% of the variance of the observation explained by the model (Fig. 2).

The *Spartina* marshes studied here are typically close to saturation, which is consistent with the modeled water content (Fig. S3). However, without a comprehensive set of water content measurements available for validation, in this communication we focus on porewater salinities.

### Sensitivity analysis

**Figure 3:**
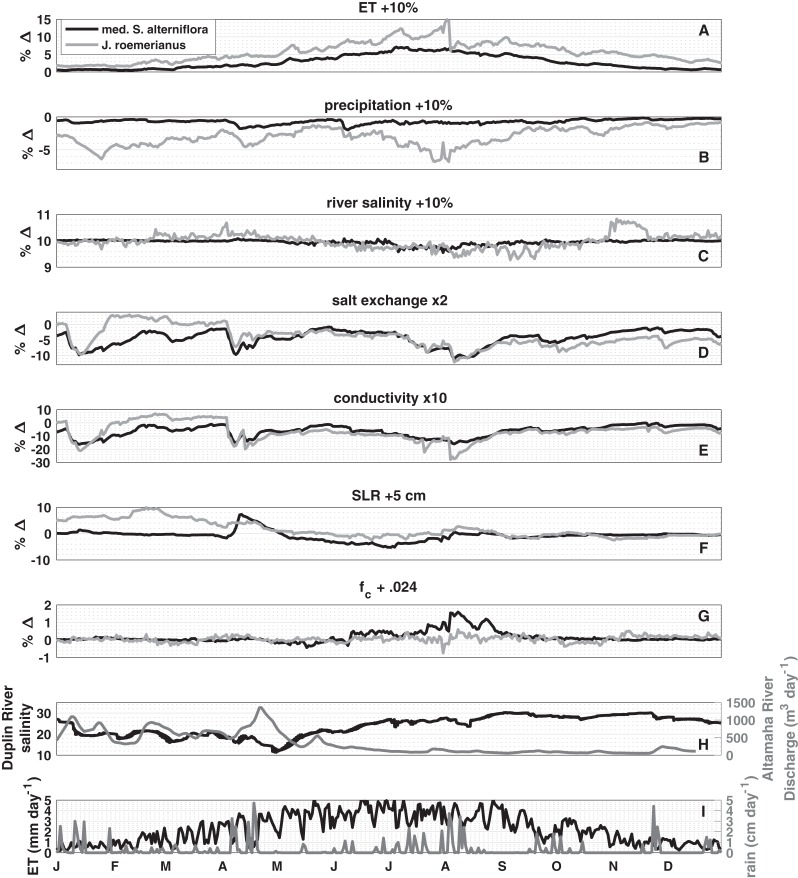
Sensitivity analysis. The median percentage change (% Δ) in porewater salinity due to a perturbation in the listed parameter or process for medium *S. alterniflora*, representing the low marsh, and *J. roemerianus*, representing the high marsh. The hydrologically average year 2014 is used in the analysis. Positive values indicate an increase in salinity. Perturbations are as follows: (A) evapotranspiration, (B) precipitation, (C) river salinity are all increased by 10%, (D) diffusivity (salt exchange) is doubled, (E) hydraulic conductivity is increased 10-fold, (F) sea level is raised by five cm, and (G) field capacity is increased by 0.024 (to 0.99 and 0.924 in low and high marsh, respectively). (H) shows tidal salinity and Altamaha River discharge and (I) daily evapotranspiration and precipitation used to force the model. Note the difference in vertical scales between panels.

A *k*-means cluster analysis of the response of the porewater salinity to changes in controlling factors (Figs. 3A–3G) revealed that the seven vegetation classes fell into two clusters (determined by the highest mean silhouette value compared to up to seven distinct clusters; and using a squared Euclidean distance metric), which represent low and high marsh environments. Medium *Spartina alterniflora* and *J. roemerianus* covered the largest surface areas in the two respective clusters and were subsequently used as representative vegetation types for the low and high marsh, respectively. The sensitivity analysis showed that increasing ET rates and Duplin River salinity increased porewater salinity. While the increase of Duplin River salinity had a spatially uniform effect on porewater salinity of 10% throughout the year (Fig. 3C), the impact of altering ET varied temporally and between high and low marsh (Fig. 3A). The positive effect peaked in the summer months and coincided with the highest ET rates throughout the year. The effect of a 10% increase in ET for the high marsh was greater than 5% from mid-April to October, with a peak of a 15% change in porewater salinity in the beginning of August, whereas the effect was less than 8% for the low marsh. Increasing precipitation by 10% had a negative effect on high marsh porewater salinity; the low marsh was nearly unaffected (Fig. 3B). Three periods of high marsh freshening stand out: mid-January to March (−5 to −7%), April (−5%), and mid-July through the beginning of August (−4% to −7%), periods during which rainfall was substantial (Fig. 3I).

Doubling salt exchange between the marsh subsurface and the overlying water lowered porewater salinity across the marsh, except from mid-January to mid-March when there was a small positive effect (<3%) in the high marsh (Fig. 3D). The magnitude of the effect depended on the difference in salt concentration in the porewater and inundating water. In the low marsh, salt exchange had the largest effect in January (−10%), April (−10%), and the beginning of August (−11%). The high marsh responded similarly: January (−10%), April (−7%), the beginning of August (−12%), which remained −4% and −9% for the remainder of the year.

Of the parameters tested, increasing hydraulic conductivity by an order of magnitude—reflecting the larger uncertainty in this parameter—resulted in the largest effects on porewater salinity in the low and high marsh (Fig. 3E). In the low marsh, increasing hydraulic conductivity always lowered porewater salinity. The largest negative effects coincided with periods of increased precipitation: January (−16%), April (−15%), and the beginning of August (−15%). The high marsh responded similarly during the same periods: January (−21%), April (−20%), and the beginning of August (−27%). From mid-February to the beginning of April there was a positive effect greater than 3% for the high marsh.

Five centimeters of sea level rise (SLR) had a positive effect on the high marsh porewater salinity from the start of the year until mid-May while there was little effect for the rest of the year (Fig. 3F). In the low marsh, during the period of increased precipitation SLR had a positive effect of 8%. From May until August SLR had a negative effect on low marsh porewater salinity, reaching a peak of −5% the beginning of July. Increasing field capacity had little effect on the high marsh (Fig. 3G), and only impacted the low marsh during June and July with a peak positive effect on 1.6% at the beginning of August.

### Three-year simulations

**Figure 4:**
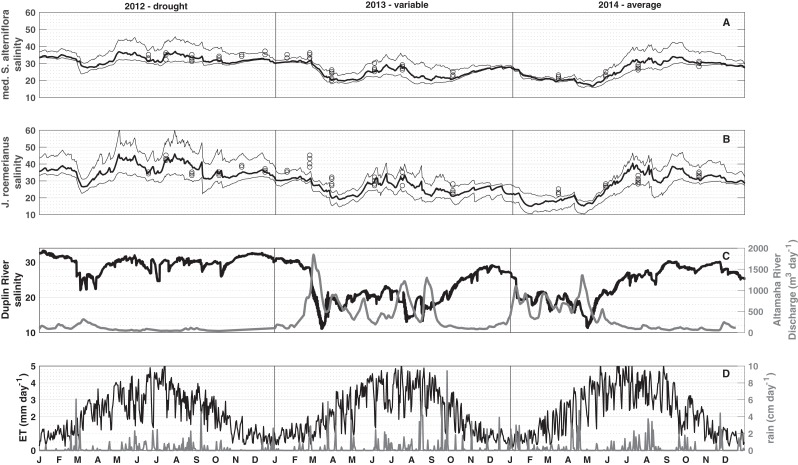
Three-year simulation. Median (bold line), and the 10^th^ and 90^th^ percentiles (thin lines) porewater salinity for 2012 (drought), 2013 (hydrologically variable), and 2014 (hydrologically average). Black circles in (A) (low marsh) and (B) (high marsh) denote the measured porewater salinities. (C) shows tidal salinity and Altamaha discharge, and (D) shows daily precipitation and evapotranspiration for medium *S. alterniflora* and *J. roemerianus* used to force the model.

To visualize spatial and temporal patterns in porewater salinities, median, and 10^th^ and 90^th^ percentiles were computed from all mesh elements vegetated by medium *Spartina alterniflora* and *J. roemerianus*, respectively (Figs. 4A and 4B). The simulation results show that the spatial variability in porewater salinity is typically small compared to the temporal variations (compare the range between the 10^th^ and 90^th^ percentiles to the temporal variation in porewater salinities within a vegetation zone in Figs. 4A and 4B; Fig. S2).

The daily mean porewater salinity in the low and high marsh follows the general pattern observed in the daily mean tidal salinity, with *R*^2^ values of 0.77 and 0.58, respectively. This signal is modulated in the short-term by precipitation events, and in the long-term by seasonal ET rates. Each spring the Duplin River salinity dropped, and a freshening of porewater salinity was observed. As spring transitioned to summer, the Duplin River salinity began to increase (Fig. 4C) along with ET rates that peaked during June–July (Fig. 4D). During May, there was typically less precipitation than in late summer (Fig. 4D), and porewater salinities began to increase to some of the highest values of the year. A main difference between the high and low marsh was the response to precipitation. From July to September, precipitation became more frequent and intense, resulting in a freshening of the porewater. This freshening effect was pronounced in the high marsh (Fig. 4B), in particular during mid-June and September 2012, July and August 2013, and July and August 2014, whereas the low marsh largely follows the salinity trend observed in the Duplin River (Fig. 4A).

Model simulations revealed substantial interannual variations in soil salinities. In 2012, decreased Altamaha River discharge caused higher than normal Duplin River salinity (Fig. 4C). Increased Duplin River salinity, together with decreased precipitation, resulted in the highest modeled salinities for the low and high marsh during the simulation. From March to mid-May, the median salinities changed from 28 to 37 for the low marsh with a 90^th^ percentile salinity of 43, and 27–43 for the high marsh with a 90^th^ percentile salinity reaching 60, starkly contrasting with the salinities during the variable/wet 2013 (holding around 20 in the low marsh and high marsh for the same period). Overall, the seasonal variability was larger in a drought year (2012) than in a variable/wet year (2013) and hydrologically average year (2014) despite 2013 and 2014 experiencing wider ranges of Duplin River salinity.

## Discussion

### Model assessment

For the entire study period salinities observed in field measurements and modeling match well in the low marsh—tall, medium, short *Spartina alterniflora*. However, there are greater deviations in areas dominated by *J. roemerianus* rather than medium *Spartina alterniflora*, which is reflected in the root mean square errors of 5.9 and 2.89, respectively (Fig. 2). The model tends to slightly overestimate *J. roemerianus* porewater salinities in the summer months—when model ET rates are highest—and underestimates salinities in the spring of 2013 and 2014 (Fig. 4B). We attribute the differences between modeled and observed salinities to uncertainties in ET, hydraulic conductivity, and, potentially, upward flow of fresher groundwater that is not accounted for in our model. In our sensitivity analysis, these factors were found to be having a substantial impact on model results. However, simulations exploring the model behavior indicated that the effect of one parameter or process could be partially counteracted by tuning another (e.g., ET and lateral flow, in regions with less frequent inundation). Thus, model parameterization relied on data available through the GCE-LTER, supplemented by the literature, rather than solely optimizing the fit of the model results to the observations in a limited area of the study domain.

Evapotranspiration rates for different cord grasses (e.g., *Spartina alterniflora*) and succulents (e.g., *Sarcocornia quinqueflora, B. maritima*, *Sarcocornia pacifica*) are computed using the Penman–Monteith equation (Hughes et al., 2001; Wang et al., 2007). However, it estimates ET under optimum soil water conditions (Allen et al., 1998), and does not consider physiological responses to salt stress, for example, reduced stomatal conductance of *J. roemerianus* when exposed to salt water treatments to minimize water loss (Touchette, 2006; Touchette et al., 2009). Decreasing summer ET rates would reduce the modeled *J. roemerianus* porewater salinities when our model tends to be on the high side (i.e., 90^th^ percentile salinities in Fig. 4B). Uncertainty in ET further arises from uncertainties in the parameterization of the Penman–Monteith equation, which depends on albedo and surface resistance. In this study, all vegetation classes were given an albedo of 0.10, due to a lack of explicitly reported values for marsh vegetation—although Moffett et al. (2010) reports minimum daily albedos of 0.073 and 0.089 during flooded and non-flooded conditions, respectively. Albedo for vegetation changes as a function of season, solar elevation angle, the available moisture (i.e. saturated vs. unsaturated), and plant cover (Shuttleworth, 1993; Hughes et al., 2001). Significant uncertainty exists in literature-reported surface resistance values for salt marshes ranging from 5 s/m (Hughes et al., 2001) to calibrated values for a flooded marsh of 67–74 s/m to 171–248 s/m for exposed marsh surfaces (Moffett et al., 2010). The substantial importance of surface resistance for estimates of ET points to the usefulness of data that constrains its value and the factors and conditions that affect it over time.

Another major source of uncertainty is the magnitude and spatial variability of hydraulic conductivity. At sites in and near the model domain, Schultz & Ruppel (2002) documented hydraulic conductivities, derived from a variety of methods (grain size analysis, falling head permeameter test, tidal pumping, and short-term pumping test), that range from on the order of 10^−9^ to 10^−4^ m/s, with hydraulic conductivity being higher in the upland than the marsh platform. Our sensitivity analysis (Fig. 3E) demonstrates that on average the low and high marsh porewater salinity decreases by 6.5%, when the hydraulic conductivity is increased by an order of magnitude, which also narrows the range of salinities within a vegetation class. Increasing high marsh hydraulic conductivity in *J. roemerianus* zones increases drainage and decreases the accumulation of salt, consistent with field observations (Bertness & Pennings, 2000).

For each sampling campaign in the 3-year period, the mean porewater salinity measured in *J. roemerianus* stands exceeded the mean salinity measured in *Spartina alterniflora*. A similar general pattern was found in the model simulations, where median *J. roemerianus* porewater salinity exceeded *Spartina alterniflora* porewater salinity the majority of the time. However, literature reports point to *J. roemerianus* commonly occurring in zones with fresher porewater than *Spartina alterniflora* (Wiegert & Freeman, 1990; Pennings, Grant & Bertness, 2005). This indicates that *J. roemerianus* may occasionally access deeper, fresher water, for example, associated with upward flow of fresh and brackish groundwater during neap tides or low precipitation periods (Wilson et al., 2015). It also points to the complex controls on plant distribution (Pennings, Grant & Bertness, 2005), severely limiting ability of models such as the one presented here to predict changes in vegetation over time. Furthermore, our model does not account for complex transport processes at the marsh-upland transition zone (Wilson et al., 2015), which can affect porewater salinity and soil moisture across ecohydrological zones (Moffett et al., 2012), or the two-phase flow dynamics that can be important for soil aeration and plant growth (e.g., Xin et al., 2013 and references therein).

### Seasonality and spatial variability

The years 2012–2014 cover a range of hydrologic conditions, yet consistent temporal and spatial patterns in porewater salinities emerge each year. In spring, when Duplin River salinity decreases, the high and low marsh salinities respond similarly. This is in agreement with the sensitivity analysis, which shows that porewater salinities follow the salinity of the flooding tide, which is lower in spring as a result of high Altamaha River discharge (Fig. 3C; Di Iorio & Castelao, 2013).

Within a vegetation class, variability is observed at any point in time (Figs. 4A and 4B), particularly from May to August; this result is in agreement with the observations reported in Richards, Pennings & Donovan (2005) and Lynes (2008). However, differences between the low and high marsh 90^th^ percentile salinity can be substantial and largely reflect differences in inundation due to elevation. The difference between the mean elevations of the two vegetation classes is 32 cm. High marsh 90^th^ percentile salinities are on average 7–17 higher than the low marsh during the months of May–August (Figs. 4A and 4B). Similarly, Morris (1995) found that increasing the plot elevation by 20 cm causes maximum salinities to nearly double.

In mid-to-late summer, precipitation events increase in frequency and intensity, and cause freshening of the porewater. This effect is much more pronounced in the high marsh. Longer periods between tidal flooding allow more precipitation to infiltrate the soil. Freshwater can be laterally transported by runoff or groundwater, a controlling factor at higher marsh elevations and the upland transition (Chapman, 1939). In mid to late fall, as ET rates decrease and precipitation events become less frequent, the porewater salinities of both low and high marsh are more controlled by Duplin River water inundation. However, our results (Fig. 3B) clearly demonstrate the sudden and profound impact that precipitation can have on high marsh porewater salinity.

### Interannual variability

Predicted interannual porewater salinities, to varying degrees, follow the trend of the Duplin River salinity, which is inversely correlated with Altamaha River flow. Comparison of the salinity measurements at GCE10 site with the Altamaha flow at Doctortown (Figs. 1 and 4C) over the duration of this study reveals that 82% of the variability in Duplin River salinity measured at GCE10 can be explained by a dependence on Altamaha discharge with a 13-day lag, similar to the findings of Di Iorio & Castelao (2013).

The marsh experiences the highest porewater salinities when the Duplin River salinity was at its maximum, which was in the drought year (2012) followed by the hydrologically average year (2014) and then the variable/wet year (2013). Peak salinities occur at different times during the 3 years. These timings are the result of differences in the balance between ET, Duplin River salinity, and precipitation. Seasonal ET rates are essentially identical for the 3 years, reaching a maximum in late-June to mid-July, but the river salinity, and magnitude and timing of precipitation is quite different between the 3 years (Fig. 4D).

The temporal patterns in precipitation and river salinity have biological implications. Physiological stress placed on *Spartina alterniflora* by increased salinity can have an impact on plant productivity (Morris, 1995; Pennings, Grant & Bertness, 2005). Photosynthate produced in the fall is transferred belowground in the winter and used to support spring growth (Lytle & Hull, 1980; Jung & Burd, 2017). Similarly, O’Donnell & Schalles (2016) report that reduced aboveground fall biomass results in lower spring biomass and growth rates. Persistent changes, for example, associated with SLR exceeding marsh accretion, also impact vegetation distribution (Craft et al., 2009).

## Conclusions

The model accurately reproduces field observations, which allows for the exploration of temporal and spatial variability in marsh porewater salinity. The obvious key process controlling porewater salinity in an intertidal salt marsh is the salinity of the inundating tide. Besides delivering the salt water that inundates the soil, tidal inundation controls the duration over which other processes can have an impact on porewater salinity. The sensitivity analysis also revealed that hydraulic conductivity can have a strong impact on porewater salinity. Precipitation and ET have their most pronounced effect on soil salinities at higher elevation, which is less frequently inundated.

Salt marshes are subject to environmental pressure due to SLR (FitzGerald et al., 2008), which may impact vegetation distribution (Craft et al., 2009). Our work shows that salt marshes can also be impacted by hydrologic changes away from the coast, as Altamaha flow is a key determinant for creek salinity and the salinity of marsh porewater at our study site, as revealed in the interannual comparison. O’Donnell & Schalles (2016) found a significant relationship between drought and marsh productivity. The spatially resolved model in this study opens the possibility to further explore the connections between soil conditions and vegetation, and to identify controls on the marsh productivity and the associated ecosystem services.

## Supplemental Information

10.7717/peerj.5911/supp-1 Supplemental Information 1 Supplemental Information. Model description and parameterization.

## Additional Information and Declarations

### Data Availability

The following information was supplied regarding data availability: https://MeileLab@bitbucket.org/MeileLab/miklesh_peerj.git.
