# Supplementary material for: Porewater salinity in a southeastern United States salt marsh: controls and interannual variation
Source: PeerJ. 2018 Nov 8;6:e5911. doi: 10.7717/peerj.5911 (PMC6230441; doi:10.7717/peerj.5911)
Supplement: Supplemental Information 1 — Model description and parameterization. [file peerj-06-5911-s001.docx]

# Supplemental Information

**Section S1. Model description**

For each one-hour time-step the mass flux for each process is computed in the following order: tidal inundation, evapotranspiration, precipitation, salt exchange, and groundwater exchange. When an element is inundated mass fluxes due to precipitation and evapotranspiration are applied to the aboveground compartment. After sinks and sources are computed for each element a pressure field is computed to determine groundwater exchange with neighboring elements.

If inundated:

|  | $\frac{dM^{H_{2}O}}{dt}=\frac{dM_{tide}^{^{H_{2}O}}}{dt}\pm\frac{dM_{GW}^{^{H_{2}O}}}{dt}+\left[-\frac{dM_{ET}^{^{H_{2}O}}}{dt}+\frac{dM_{rain}^{^{H_{2}O}}}{dt}\right]^{aboveground}$ |  |
| --- | --- | --- |

|  | $\frac{dM^{salt}}{dt}=\frac{dM_{tide}^{^{salt}}}{dt}\pm\frac{dM_{SaltExchange}^{^{salt}}}{dt}\pm\frac{dM_{GW}^{^{salt}}}{dt}$ |  |
| --- | --- | --- |

else:

|  | $\frac{dM^{H_{2}O}}{dt}=-\frac{dM_{ET}^{^{H_{2}O}}}{dt}+\frac{dM_{rain}^{^{H_{2}O}}}{dt}-\frac{dM_{drainage}^{^{H_{2}O}}}{dt}\pm\frac{dM_{GW}^{^{H_{2}O}}}{dt}$ |  |
| --- | --- | --- |

|  | $\frac{dM^{salt}}{dt}=-\frac{dM_{drainage}^{^{salt}}}{dt}\pm\frac{dM_{GW}^{^{salt}}}{dt}$ |  |
| --- | --- | --- |

In brief, the amount of water added by rain is calculated as the product of the amount of rain over a time interval ($R$), the element surface area ($A_{sfc}$) and the freshwater density ($\rho_{fw}$), $\frac{dM_{rain}^{H_{2}O}}{dt}=R*A_{sfc}*\rho_{fw}$. It is added to the subsurface if pore space is available and subsequently added to the surface water.

The amount of fluid added or removed via tidal surface exchange is computed as the product of the change in tidal elevation ($\Delta z_{tide}$ ), the surface area of the element and the water density ($\rho_{sw}$), if the element is connected to the tidal channel at the current water depth: $\frac{dM_{tide}}{dt}={\Delta z_{tide}*A}_{sfc}*\rho_{sw}$. This mass flux is then split between H_2_O ($\frac{dM_{tide}^{H_{2}O}}{dt}$) and salt ($\frac{dM_{tide}^{H_{2}O}}{dt}$), based on the mass fraction of salt in the fluid.

The amount of fluid added or removed through groundwater flow is computed as the product of a flow velocity, the exchange area between neighboring elements ($A_{xch};$see Fig. S3), the fluid density and the porosity. Active if the water content exceeds the residual water content, the flow velocity reflects the product of the hydraulic conductivity and the pressure gradient ($\frac{\Delta P}{\Delta x})$, so that $\frac{dM_{GW}}{dt}=-\frac{K}{g}*\frac{\Delta P}{\Delta x}{*A}_{xch}*\phi$. The code implementation further also accounts for surface topography, such as creek banks, allowing for seepage. The mass flux is then split between H_2_O ($\frac{dM_{GW}^{H_{2}O}}{dt}$) and salt ($\frac{dM_{GW}^{salt}}{dt}$), based on the mass fraction of salt in the fluid.

The amount of water removed through evaporation, $\frac{dM_{ET}^{H_{2}O}}{dt}$, is computed as the product of the element surface area and the freshwater density and the hourly evapotranspiration rate. The latter is computed following the procedure given in Allen et al. (1998).
$\frac{dM_{drainage}}{dt}$, the amount of fluid removed through drainage, is calculated as the product of the fluid density, the surface area, the porosity and the drainage velocity. The latter is computed as the product of the saturated hydraulic conductivity ($K_{s})$ and the head gradient ($\frac{\Delta h}{d_{creek}})$ between the element and the nearest tidal creek, $\frac{dM_{drainage}}{dt}=K_{s}*\frac{\Delta h}{d_{creek}}$. This mass flux is then split between water ($\frac{dM_{drainage}^{H_{2}O}}{dt}$) and salt ($\frac{dM_{drainage}^{salt}}{dt}$), based on the mass fraction of salt in the fluid.

The amount of salt exchanged between surface and subsurface boxes is computed as the product of the diffusion coefficient ($D)$, the concentration difference between above and belowground boxes ($\Delta C)$, the element surface area and the porosity, divided by the exchange length scale ($d_{xch})$, $\frac{dM_{SaltExchange}^{salt}}{dt}=-D\frac{\Delta C}{d_{xch}}*A_{sfc}*\phi$.


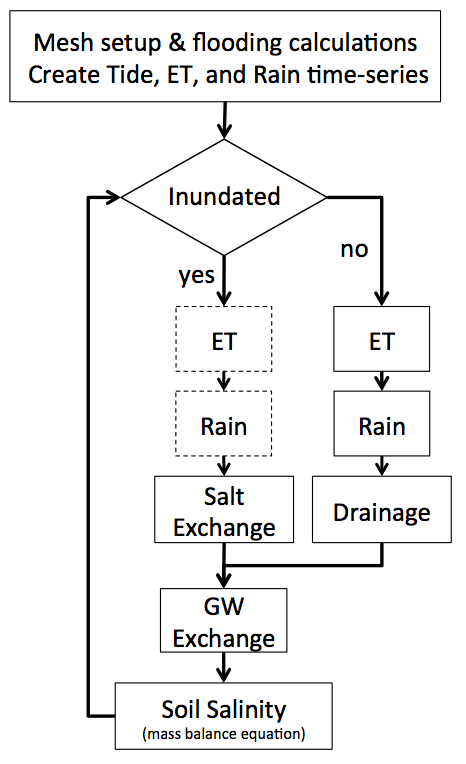


Figure S1. Flow chart of the soil model used in this study. Dashed box indicates active aboveground compartment process.

Model application thus provides soil pore water salinities (and water content) as a function of space and time. Examples are shown in Figures S2 and S3.


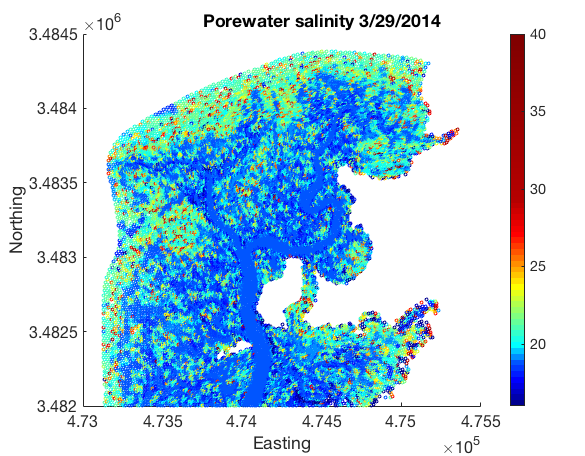


Figure S2. Snapshot of the simulated porewater salinities. The centroid of each mesh element in the upper Duplin is represented by a circle.


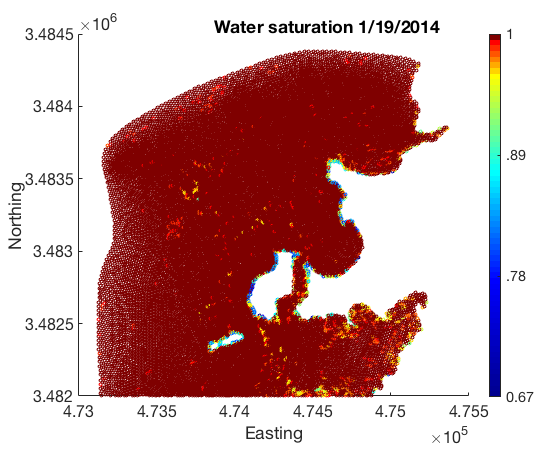


Figure S3. Snapshot of the simulated water saturation. The centroid of each mesh element in the upper Duplin is represented by a circle.

*Mesh geometries*

Prior to running the model, mesh geometries are computed. For each element of the mesh, the following characteristics were computed: surface, element centroid, angle between centroids and normal, the area between neighboring sediment compartments, and above and belowground flux area.


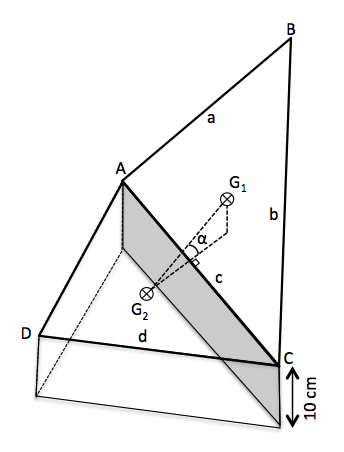


Figure S4. Diagram to demonstrate surface area (e.g., triangle ABC), element centroid (G), angle between centroids and normal (α), area between neighboring sediment compartments (shaded area) calculations.

Surface area (Δ) [L^2^] is calculated using Heron’s formula:

|  | $\Delta=\sqrt{s\left(s-a\right)\left(s-b\right)\left(s-c\right)}$ |  |
| --- | --- | --- |

where

|  |  |  |
| --- | --- | --- |
|  | $s=\frac{\left(a+b+c\right)}{2}$ |  |

with side lengths *a*, *b*, and *c*.

The centroid (*G*) of the triangular element is calculated from the node locations *A* = (*x_a_*, *y_a_*, *z_a_*), *B* = (*x_b_*, *y_b_*, *z_b_*), and *C* = (*x_c_*, *y_c_*, *z_c_*):

|  | $G=\frac{1}{3}\left(A+B+C\right)=\left(\frac{1}{3}\left(x_{a}+x_{b}+x_{c}\right),\frac{1}{3}\left(y_{a}+y_{b}+y_{c}\right),\frac{1}{3}\left(z_{a}+z_{b}+z_{c}\right)\right)$ |  |
| --- | --- | --- |

The flux angle (α) is the angular difference between two centroids’ angle (*θ_G_*) and the angle normal component ($\theta_{\hat{N}}$) of the common side. The angle between two centroids is:

|  | $\theta_{G}=\arctan\left(\frac{y_{G2}-y_{G1}}{x_{G2}-x_{G1}}\right)$ |  |
| --- | --- | --- |

and the normal component angle of the common side:

|  | $\theta_{\hat{N}}=\arctan\left(-\frac{x_{c}-x_{a}}{y_{c}-y_{a}}\right)$ |  |
| --- | --- | --- |

gives the flux angle as:

|  |  |  |
| --- | --- | --- |

The area between neighboring sediment compartments is a side length of an element multiplied by the pedon depth (shaded area in Fig. S4).

**Section S2. Critical-flooding**

The marsh topography is complex, with numerous tidal creeks of varying sizes, levees separating the creeks from the low marsh, and small depressions scattered throughout the marsh. As a result, the tidal level at which locations of the marsh flood does not necessarily coincide with the land elevation. To determine the tide level at which a marsh parcel floods, the algorithm presented in Barnes et al. (2014) was implemented.

With a user defined resolution (e.g., 0.01 m) a vector of flooding elevations is created that is used to determine the critical-flood elevation of mesh nodes or element centroids. The algorithm determines an element that has an elevation just below lowest low tide—determined from hydrographic sonde data—and uses this as the starting element, putting it in a queue. The queue is a list of elements that get tested at each flooding elevation. Pulling the first element from the queue, the algorithm test to see if the element elevation is less than the flooding elevation. If it is, the element is assigned a critical-flood elevation equal to the current flooding elevation. Neighboring elements of the flooded element are added to the queue and the element at the front of the queue is pulled. The process of pulling elements from the queue continues until the queue is empty. When queue is empty the algorithm moves to the next highest flooding elevation and returns to the original element. The whole processes is repeated at the new flooding elevation, however elements that were previously flooded retain their assigned critical-flood elevation.


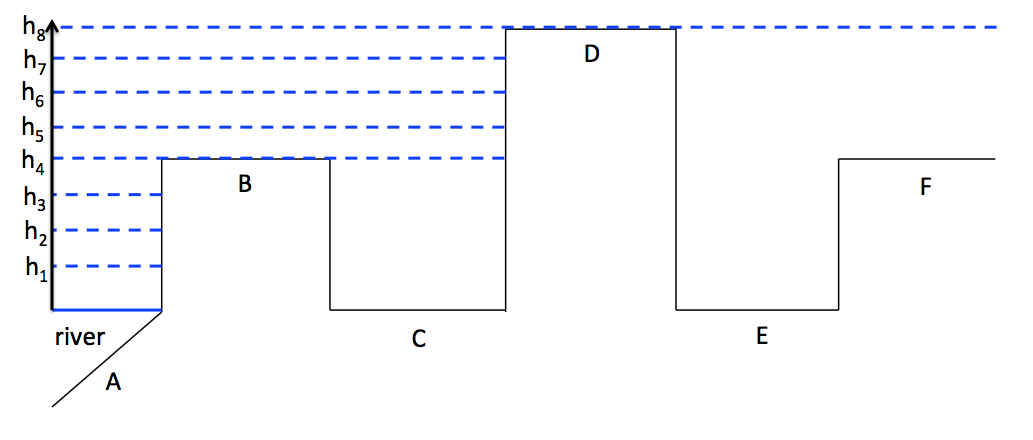


Figure S5. Diagram visualizing the critical flood process in cross section; the actual implementation takes into consideration the actual connectivity (i.e. flow around regions of higher elevation)

A vector of elevations is created (h_1_ – h_8_; Fig. S5) and element A is put into a queue. The algorithm keeps testing elevations until element B becomes flooded at h_4_. Neighbors of element B are added to the queue, which in this case is element C. Since the elevation of element C is less that h_4_, element C is assigned a critical flood elevation of h_4_. Now neighbors of element C are added (here D). D is not flooded at this elevation, so the algorithm starts over at element A. The tidal elevations are increased until all elements are assigned a critical-flood elevation. For example, element E and F would have a critical flood elevation of h_8_.

## Section S3. Estimating the hydraulic conductivity

When the rate of precipitation exceeds the infiltration rate of a soil runoff occurs. Runoff is more likely to occur in an initially wet soil than an initially dry soil, because in an initially dry soil infiltration is not only driven by gravity but also by suction (Hillel, 1998); as the soil profile becomes wetted, suction becomes negligible. Whether soil is saturated or unsaturated, when the precipitation rate exceeds the infiltration rate of the soil runoff occurs. Runoff can be described using the curve number (CN) method, an empirical approach developed by the USDA’s (then) Soil Conservation Service in the early 1950’s (Garen & Moore, 2005). Runoff (Q) is computed as:

|  | $Q=\frac{\left(P-I\right)^{2}}{\left(P-I\right)+S}$ $P>I$  |  |
| --- | --- | --- |

with

|  | $I=\lambda S$ |  |
| --- | --- | --- |

so that

|  | $Q=\frac{\left(P-0.2S\right)^{2}}{P+0.8S}$ |  |
| --- | --- | --- |

and where S is given by

|  | $S=\frac{2540}{CN}-25.4$ |  |
| --- | --- | --- |

where *Q* is actual direct runoff [cm], *P* is total rainfall [cm], *I* is the initial abstraction [cm], *λ* is the initial abstraction ratio, *S* is watershed storage [cm], and *CN* is the dimensionless curve number. The CN and λ are the only two parameters used in the method to determine the approximate amount of direct runoff from a rainfall event. The CN ranges from 0 for fully permeable to 100 for impermeable surfaces. Curve number tables approximate the *CN* of marshes to be 90 during dry periods and 98 during wet periods (Suphunvorranop, 1985). Classically, λ was set to 0.2, but more recent studies (e.g., Woodward et al., 2003) have found this value to be approximately 0.05. To determine a representative, spatially averaged hydraulic conductivity a mechanistic method of computing runoff was compared to the empirical method described above.

To that end, Marsh Landing precipitation data from 2002 – 2011 was binned by frequency of rainfall intensity (cm per 15-minute increment) and total daily precipitation. This analysis revealed that precipitation commonly occurs at low intensity that extends for longer duration for low total daily precipitation and a few intense events contribute to days with high daily precipitation. For a given daily rainfall, 1000 Monte Carlo simulations were then performed with rainfall selected from the observed frequency distribution of rainfall intensities. Infiltration was then computed as the minimum of the precipitation and saturated hydraulic conductivity. The mean and standard deviation for the event-based mechanistic infiltration estimate was computed for a range of hydraulic conductivities and plotted together with infiltration estimated by the curve number method (Fig. B1). This analysis suggests that curve number values of 94 produces a hydraulic conductivity of 2x10^-7^ m s^-1^ (Fig. S6), which is in reasonable agreement with conductivities reported in Schultz and Ruppel (2002) and Wang et al. (2007). In the high marsh and upland fringe the soils become sandier and are infrequently inundated so a lower CN, leading to a higher estimate of the hydraulic conductivity, would reflect the hydrologic state of the soil.

Figure S6. Mechanistically computed infiltration (circles with error bars) and CN computed infiltration (—).

## Section S4. Code and data availability

The matlab code, example input files and measured salinity data can be downloaded from <https://MeileLab@bitbucket.org/MeileLab/miklesh_peerj.git>

REFERENCES

Barnes R, Lehman C, Mulla D. 2014. Priority-flood: An optimal depression-filling and watershed-labeling algorithm for digital elevation models. *Computers & Geosciences,* 62:117-127.

Garen DC, Moore DS. 2005. Curve number hydrology in water quality modeling: uses, abuses, and future directions. *Journal of the American Resources Association,* 41:377-388*.*

Hillel D. 1998. *Environmental soil physics: Fundamentals, applications, and environmental considerations*. Elsevier.

Schultz G, Ruppel C. 2002. Constraints on hydraulic parameters and implications for groundwater flux across the upland–estuary interface. *Journal of Hydrology*, 260(1):255-269.

Suphunvorranop T. 1985 A guide to SCS runoff procedures, Technical Publication SJ 85-5.

Wang H, Hsieh YP, Harwell MA, Huang W. 2007. Modeling soil salinity distribution along topographic gradients in tidal salt marshes in Atlantic and Gulf coastal regions. *Ecological Modelling,* 201(3–4):429-439.

Woodward DE, Hawkins RH, Jiang R, Hjelmfelt Jr AT, Van Mullem JA, Quan QD. 2003. Runoff curve number method: examination of the initial abstraction ratio. In *World Water & Environmental Resources Congress 2003*. 1-10.
